# Enhancing Thermoelectric Properties through Control of Nickel Interstitials and Phase Separation in Heusler/Half-Heusler TiNi_1.1_Sn Composites

**DOI:** 10.3390/ma11060903

**Published:** 2018-05-28

**Authors:** Emily E. Levin, Francesca Long, Jason E. Douglas, Malinda L. C. Buffon, Leo K. Lamontagne, Tresa M. Pollock, Ram Seshadri

**Affiliations:** 1 Materials Department, University of California, Santa Barbara, CA 93106, USA; emilylevin@mrl.ucsb.edu (E.E.L.); francescalong2021@u.northwestern.edu (F.L.); jasonedouglas@gmail.com (J.E.D.); mandibuffon@gmail.com (M.L.C.B.); lamontagne@mrl.ucsb.edu (L.K.L.); pollock@engineering.ucsb.edu (T.M.P.); 2 Materials Research Laboratory, University of California, Santa Barbara, CA 93106, USA

**Keywords:** Heusler, TiNiSn, TiNi_2_Sn, point defect, thermoelectric, phonon scattering

## Abstract

Thermoelectric devices, which allow direct conversion of heat into electrical energy, require materials with improved figures of merit (zT) in order to ensure widespread adoption. Several techniques have been proposed to increase the zT of known thermoelectric materials through the reduction of thermal conductivity, including heavy atom substitution, grain size reduction and inclusion of a semicoherent second phase. The goal in these approaches is to reduce thermal conductivity through phonon scattering without modifying the electronic properties. In this work, we demonstrate that Ni interstitials in the half-Heusler thermoelectric TiNiSn can be created and controlled in order to improve physical properties. Ni interstitials in TiNi${}_{1.1}$Sn are not thermodynamically stable and, instead, are kinetically trapped using appropriate heat treatments. The Ni interstitials, which act as point defect phonon scattering centers and modify the electronic states near the Fermi level, result in reduced thermal conductivity and enhance the Seebeck coefficient. The best materials tested here, created from controlled heat treatments of TiNi${}_{1.1}$Sn samples, display zT = 0.26 at 300 K, the largest value reported for compounds in the Ti–Ni–Sn family.

## 1. Introduction

Thermoelectric materials, which convert between thermal and electric energy through solid state phenomena, have the potential to harvest waste heat, therefore reducing energy consumption and greenhouse gas production [1]. While thermoelectrics currently have a variety of niche uses such as thermoelectric radioisotope generators in space probes, the widespread adoption of thermoelectric technology awaits more efficient devices, whose efficiency depends on the figure of merit (zT) of the materials employed [2,3]. The figure of merit is given by the equation $zT=[S^{2}/\left(\rho \;\kappa \right)]T$ where *S*, ρ, κ and *T* are the Seebeck coefficient, electrical resistivity, thermal conductivity and temperature, respectively. These properties are interrelated, frequently making it difficult to improve the overall zT [1,4]. For example, increasing the electrical conductivity (decreasing ρ) will increase thermal conductivity due to the electronic contribution to κ. However, the lattice contribution to κ can be reduced independently by the insertion of phonon scattering centers [3,5,6]. Engineering advanced materials through techniques to enhance phonon scattering across different length scales such as doping and heavy atom substitution [7,8,9,10,11], micro-/nano-structuring through grain size reduction [3,10,12,13] and phase separation [14,15,16,17,18,19,20,21,22] has been shown to increase the figure of merit of known thermoelectric materials. State-of-the-art thermoelectric materials such as Bi${}_{2}$Te${}_{3}$ and AgSbTe${}_{2}$ exhibit values of zT ≈ 1–1.5 at their optimal operation temperature [1,23,24,25,26].

**Figure 1:**
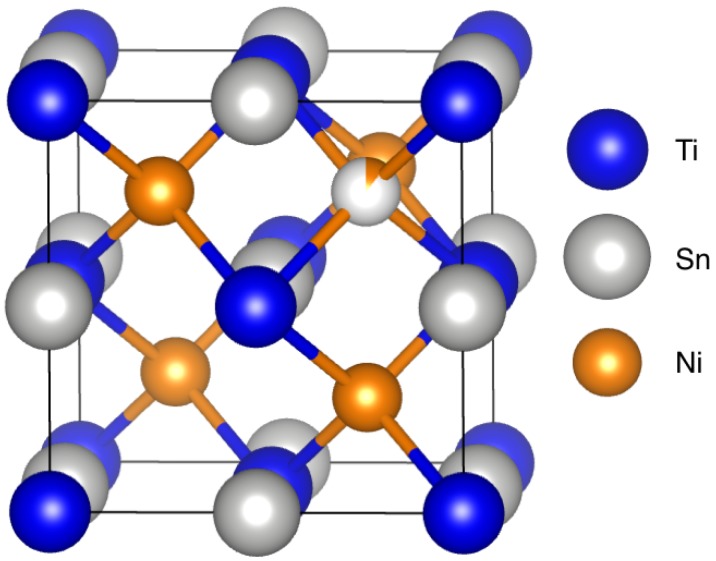
Crystal structure of half-Heusler TiNiSn in the $F\bar{4}3m$ space group. Excess Ni occupies vacant tetrahedral sites, as shown by the partially-occupied Ni interstitial. All four of the vacant tetrahedral sites are filled in the full-Heusler, in the space group $Fm\bar{3}m$.

Half-Heusler materials are promising for middle-to-high temperature range (600 K–900 K) thermoelectric applications due to their intrinsically high power factor ($S^{2}/\rho$), despite their high thermal conductivity. Douglas et al. have shown that hierarchical microstructural engineering through the inclusion of a semicoherent second phase in TiNiSn decreases thermal conductivity by scattering phonons at multiple length scales [3,10,15,27]. In this compound, the addition of excess Ni leads to phase separation between the full- and half-Heusler upon solidification. The two phases are immiscible at moderate temperatures. However, the phase diagram presented by Verma et al. suggests that both regions of phase separation and phase solubility can be accessed at the composition TiNi${}_{1.1}$Sn [28]. At high temperatures, the excess Ni fills the tetrahedral voids in the half-Heusler crystal structure, shown in Figure 1 [27,29,30]. Hazama et al. have shown that TiNi${}_{1+x}$Sn follows Vegard’s law, with the lattice parameter expanding linearly with the addition of nickel [31,32]. In this work, heat treatments are used to trap these Ni interstitials, and the properties are analyzed as a function of the prevalence of Ni interstitials and the microstructure.

Nickel interstitials act as point defect scattering centers for phonons, reducing the thermal conductivity through alloy scattering [32,33,34]. In addition, these interstitials modify the electronic structure near the Fermi energy, providing “in-gap states”, which affect the Seebeck coefficient and electrical resistivity [35,36]. Miyamoto et al. observed these “in-gap states” using X-ray photoemission spectroscopy on stoichiometric TiNiSn and attributed these states to the atomic disorder present when Ni sits on the vacant site. The variability in reported zT of the half-Heusler TiNiSn is likely due to the presence of varying amounts of Ni interstitials, which are highly dependent on the processing conditions [30,33,35]. In this contribution, the disorder is built in by using heat treatments to modify the occupancy of Ni on the vacant site rather than the use of additional alloying elements. As shown here, the processing conditions and thermal history of TiNiSn compounds are extremely important for the prevalence of Ni-interstitials and microstructure, which have large effects on the physical properties. By contributing these insights on the relationship between the processing, structure and thermoelectric performance of TiNiSn, this work enables both the understanding of fundamental concepts behind defect-engineering and the development of high performance thermoelectric materials.

## 2. Experimental Details

**Figure 2:**
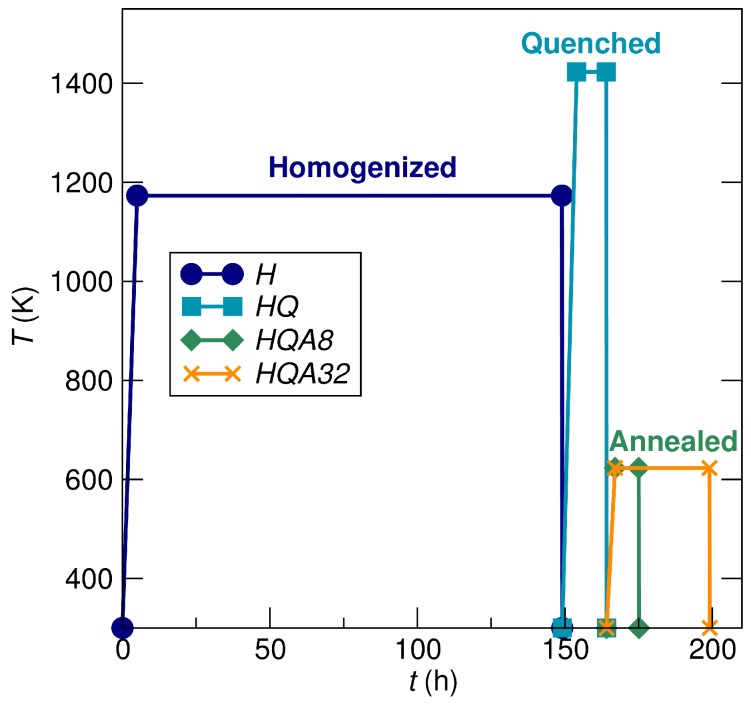
Nature of heat treatments employed in this study. Four samples were prepared: *H*, *HQ*, *HQA8* and *HQA32* (*H*, homogenized; *Q*, quenched; *A*, annealed). With the exception of the final annealing for *HQA8* and *HQA32*, heat treatments were applied to samples simultaneously.

Four TiNi${}_{1.1}$Sn samples were melted utilizing a Crystalox MCGS5 levitation melting system with a water-cooled copper crucible under an Ar atmosphere. Charges of approximately 12 g were formed from a stoichiometric ratio of TiNi${}_{1.1}$Sn from elemental sources: Ti wire (99.7%, Sigma Aldrich, St. Louis, MO, USA), Ni foil (99.9%, Sigma Aldrich) and Sn shot (99.8%, Sigma Aldrich). As visualized in Figure 2, heat treatments were applied to each sample. The homogenized (*H*) sample was held at 1173 K for 144 h (6 d), after which the sample was air-quenched. The homogenized-quenched (*HQ*) sample was homogenized with *H* and was subsequently brought to 1423 K for 6 h from which it was air-quenched. The homogenized-quenched-annealed-8 h (*HQA8*) sample followed the same heat treatment as *H* and *HQ* and was then annealed for 8 h at 623 K. Similarly, the homogenized-quenched-annealed-32 h (*HQA32*) sample was heat-treated with the other samples, but annealed for 32 h at 623 K. All heat treatments were conducted by wrapping the samples in Ta foil and sealing in a fused silica ampoule under vacuum. Samples were sectioned by a diamond saw for the experiments, including a piece to grind into a powder for synchrotron X-ray diffraction, a piece for scanning electron microscopy and a bar for physical property measurements, approximately 8 mm× 3 mm× 3 mm.

Synchrotron X-ray diffraction (XRD) data were acquired at the 11-BM beamline at the Advanced Photon Source at Argonne National Lab. Measurements were conducted at 295 K and run at a modified wavelength (λ = 0.460461 Å) to reduce Sn absorbance. Rietveld refinement analysis was completed on diffraction data using TOPAS [37]. Crystal structures were visualized using VESTA [38].

Samples of *H*, *HQ* and *HQA32* were prepared for microstructure evaluation using scanning electron microscopy by mounting a piece from the bulk samples in epoxy and polishing with a diamond suspension down to 0.25 μm. Studies were conducted on an FEI XL30 Sirion FEG scanning electron microscope (SEM, FEI, Hillsboro, OR, USA) equipped with a backscattered-electron detector (BSE, FEI) and energy dispersive X-ray spectrometer (EDS, EDAX, Newark, NJ, USA), enabling phase observation and composition determination. Values for the composition were averaged over several EDS point measurements. Electron transparent lamellae were prepared using a focused ion beam (FIB, Helios, FEI) and characterized using an FEI Tecnai G2 Sphera transmission electron microscope (TEM, FEI).

Electrical transport properties (Seebeck coefficient and electrical resistivity) were evaluated on an ULVAC ZEM-3 instrument (ULVAC, Methuen, MA, USA) under a partial He atmosphere. Measurements were conducted at 310 K. Thermal conductivity measurements were conducted on a Quantum Design Physical Property Measurement System (PPMS, Quantum Design, San Diego, CA, USA) utilizing the Thermal Transport Option (TTO). At elevated temperatures, the Ni interstitials that were trapped in the half-Heusler structure are able to diffuse, changing the nickel distribution. Due to the effect of high temperature measurements on samples, measurements were taken between 300 K and 310 K [28].

## 3. Results and Discussion

### 3.1. Structural Characterization

**Figure 3:**
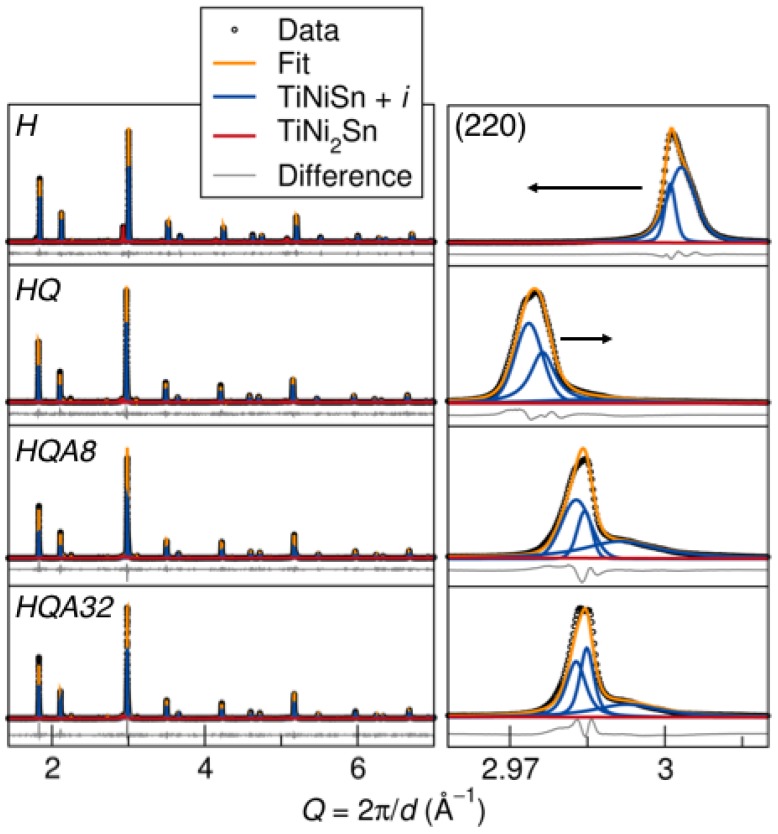
Rietveld refinement of synchrotron X-ray diffraction data for each sample, showing the individual contributions from each major phase present. The enhanced view of the (220) peak (right panel) shows asymmetry that must be fit using multiple phases with Ni-interstitials. The shifting of these peaks implies a larger lattice parameter of the TiNiSn + *i* phase in the *HQ* sample, consistent with the theory that there are more kinetically-trapped Ni interstitials due to the heat treatment.

The high signal-to-noise ratio of synchrotron X-ray diffraction (SXRD) enables precise analysis of half-Heusler and Heusler phase fractions using Rietveld refinement, as well as identification of secondary phases. Small amounts (<3%) of Sn and Sn${}_{5}$Ti${}_{6}$ were identified. The fine *Q*-space resolution of this technique allows for the evaluation of accurate lattice parameters and the observation of the asymmetry in peaks corresponding to different lattice parameters in the half-Heusler phase due to changing Ni content. Refinements of SXRD are shown in Figure 3, including individual phase contributions from major phases.

The half-Heusler TiNiSn, in the space group $F\bar{4}3m$, consists of four interpenetrating fcc sublattices, one of which is vacant (Figure 1). The covalent nature of the [NiSn] sublattice is emphasized by viewing this as a zinc blende sublattice, with Ti occupying octahedral voids [39]. This structure containing 18 valence electrons is valence precise, with four valence electrons per atom in the [NiSn]${}^{4-}$ zinc blende network, making this compound semiconducting [40]. Excess Ni incorporated into the structure via heat treatments occupies the vacant tetrahedral sites or the unoccupied sublattice [29]. A peak shift toward lower *Q* corresponds to a larger lattice parameter, implying a higher occupancy of Ni interstitials.

**Figure 4:**
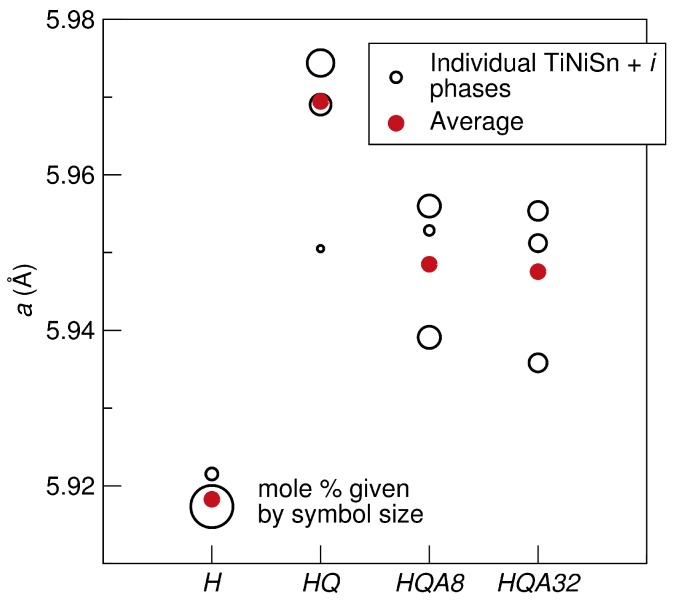
Lattice parameters for each of the half-Heusler phases fit using Rietveld refinement. Symbol size of the open circles corresponds to the mole percent of each contribution. The weighted average lattice parameter of the half-Heusler phases in each sample is given by the red filled circles and is maximized for the HQ sample where the most Ni interstitials are trapped in the half-Heusler structure. Annealed samples have a large range of lattice parameters, and the distribution has shifted to a lower lattice parameter than the HQ sample.

The enhanced view of the half-Heusler (220) peak in Figure 3 illustrates that multiple TiNi${}_{1+x}$Sn phases with lattice parameters varying ≤0.4% are necessary to fit the asymmetric peak shape [27]. This peak corresponds only to the half-Heusler, with no contributions from other phases. Fitting this asymmetry with multiple half-Heusler phases with different lattice parameters indicates an inhomogeneous distribution of Ni interstitials, rather than distinct half-Heusler phases. The occupancy of the second nickel site could not be fit due to the peak overlap; however, a larger lattice parameter implies a higher nickel content. The asymmetry is present even in the homogenized (*H*) sample, meaning there are Ni interstitials present even in what should be pure TiNiSn. This is observed in stoichiometric TiNiSn and likely leads to a wide range of properties measured on different samples [27]. The weighted average lattice parameter of the half-Heusler phases (hH $a_{av}$) in each sample (see Table 1) gives an indication of the overall amount of Ni interstitials trapped in the half-Heusler structure. This can be qualitatively seen by the peak shifting in *Q* space and is given in Figure 4. The homogenized sample (*H*) has phase separated, and the lattice parameter of the half-Heusler phase is low. For the composition TiNi${}_{1.1}$Sn at equilibrium, 10 mol% of the full-Heusler phase is expected, but the homogenized sample has a larger than nominal percentage of the full-Heusler phase. After treatment at high temperature and quenching (HQ), Ni interstitials are trapped, and the average lattice parameter is maximized. In this sample, there is no half-Heusler without Ni interstitials. This treatment is accompanied by a decrease in the fraction of full-Heusler, given in Table 1. The low temperature annealing (HQA8 and HQA32) drives the system back towards equilibrium, and the lattice parameter decreases as Ni interstitials diffuse out of the half-Heusler. The tails on the peaks in the annealed samples are much more pronounced, signifying a wider Ni distribution. The full-Heusler peak is also very broad in these samples, perhaps due to inhomogeneity or strain from the half-Heusler matrix.

### 3.2. Microscopy

**Figure 5:**
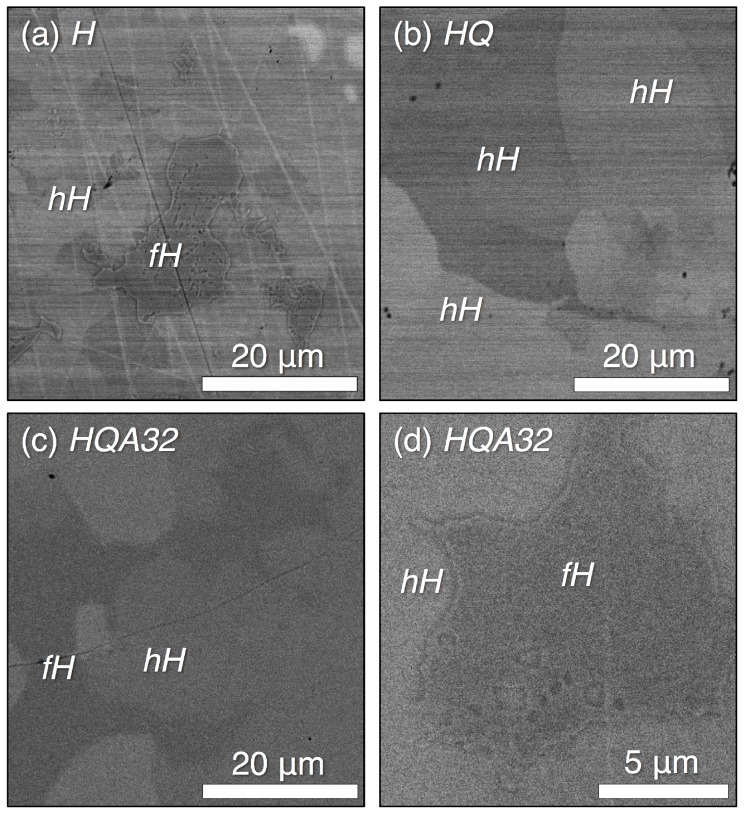
BSE SEM micrographs show the orientation and phase contrast in samples (**a**) *H*, (**b**) *HQ* and (**c**,**d**) *HQA32*, enabling analysis of microstructure evolution. (a) The homogenized sample contains separate TiNiSn and TiNi${}_{2}$Sn regions; (b) after quenching from a high temperature, we do not observe the Heusler phase, but homogeneous half-Heusler, which according to EDS, contains excess Ni; (**c**) low temperature annealing reintroduces the Heusler phase, as detailed in (d).

**Figure 6:**
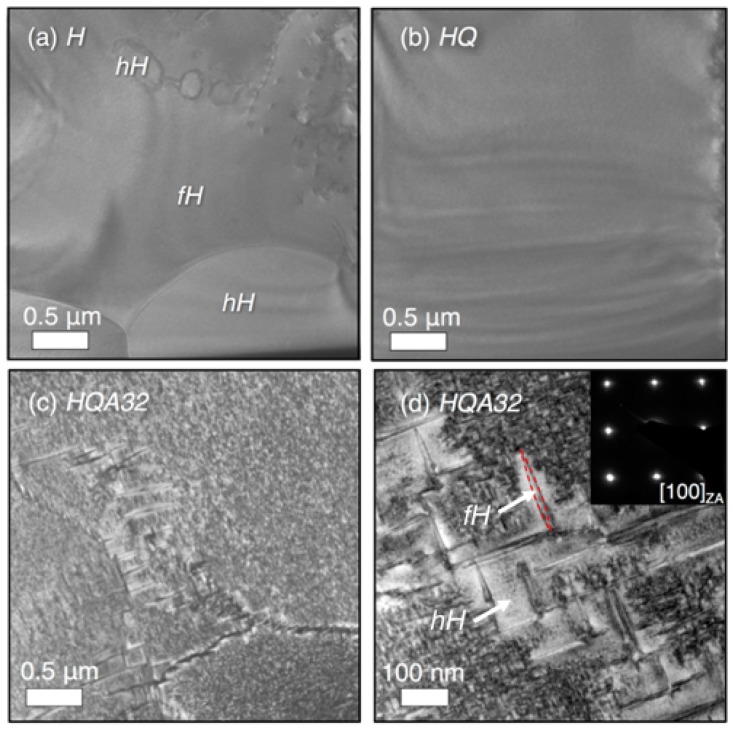
Transmission electron micrographs showing microstructural evolution in samples (**a**) *H*, (**b**) *HQ* and (**c**,**d**) *HQA32*. (a) The homogenized sample contains separate TiNiSn and TiNi${}_{2}$Sn regions on the micron scale; (b) quenching from a high temperature again reveals a single phase with excess Ni; (c,d) bright field images (along the [100] zone axis, shown by the inset in (d)) of *HQA32* show the Heusler phase precipitates out at the nanoscale, forming along 〈100〉 directions of the half-Heusler matrix, with larger precipitates forming along low angle grain boundaries. One of these larger precipitates is outlined in red. The mottled contrast in (c,d) is due to smaller scale precipitates.

The series of micrographs shown in Figure 5 was collected by SEM using a back-scattered electron (BSE) detector in order to evaluate the evolution of the microstructure with heat treatment. BSE images show *Z*-contrast, allowing us to visually distinguish between phases and to a lesser extent orientation contrast due to electron channeling [41]. Local EDS measurements elucidate the chemical composition of each phase. The homogenized sample, shown in Figure 5a, shows phase separation between the full- and half-Heusler phases, with the compositions Ti${}_{0.98(1)}$Ni${}_{1.78(2)}$Sn${}_{1.00(1)}$ and Ti${}_{0.97(1)}$Ni${}_{1.03(1)}$Sn${}_{1.00(1)}$, respectively. The TEM micrograph in Figure 6a confirms the micron-scale phase separation of the full- and half-Heusler after the homogenization treatment. The semicoherent interface between half-Heusler precipitates and the Heusler phase shows evidence of misfit dislocations [28]. The 3% lattice mismatch between the full- and half-Heusler phases produces strain fields extending in to each phase, which contribute to phonon scattering [27]. After the high temperature treatment and quenching (*HQ*), the measured composition (SEM EDS) is uniformly Ti${}_{0.98(1)}$Ni${}_{1.13(1)}$Sn${}_{1.00(1)}$, indicating the presence of excess Ni in the half-Heusler. The contrast in this image arises due to grain orientation. While XRD indicated the presence of some Heusler phase in sample *HQ*, only one phase is identified by SEM (Figure 5b) or TEM (Figure 6b), which is homogeneous, displaying only bend contours. Upon low temperature annealing *HQA32*, we again identify the Heusler phase, as highlighted in Figure 5c,d. These large-scale Heusler precipitates likely formed during the initial solidification and never underwent complete dissolution into the half-Heusler matrix during the high temperature heat treatment. TEM is used to elucidate the reformation of the Heusler from the supersaturated TiNi${}_{1.1}$Sn phase.

Low temperature annealing of the homogeneous TiNi${}_{1.1}$Sn phase forms nanoscale Heusler precipitates with a high aspect ratio, shown in Figure 6c. This microstructure has been observed by Verma et al. [28]. These precipitates are semicoherent with the half-Heusler matrix, forming with a cube-on-cube orientation relationship along the 〈100〉 directions of the half-Heusler. Larger precipitates form along low angle grain boundaries, likely due to increased diffusion of Ni along the grain boundary. This could also be to accommodate strain at the grain boundary. The light regions around these larger precipitates are depleted of excess Ni, and so, we do not see smaller precipitates in these regions. Heusler precipitates in the bulk of the half-Heusler grains are <50 nm. The nanostructuring as a result of these heat treatments leads to enhanced thermoelectric properties compared to the homogenized sample due to phonon scattering from a second phase on multiple length scales.

### 3.3. Physical Properties

**Figure 7:**
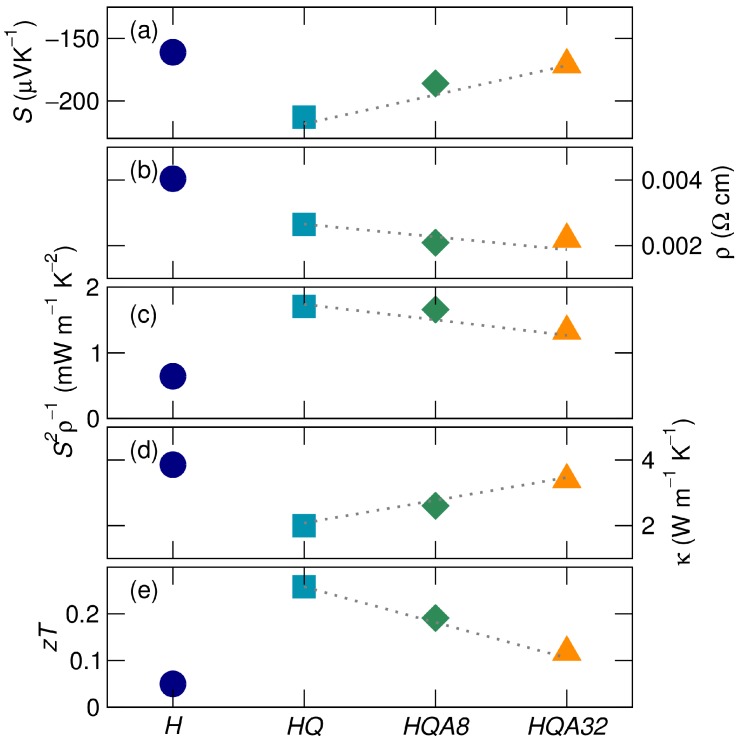
Thermoelectric physical properties for heat-treated samples with treatment identified on the *x*-axis. (**a**) The Seebeck coefficient is greatly enhanced for sample *HQ* and reduces with subsequent low temperature annealing; (**b**) resistivity subtly decreases for *HQ*, and limited change with annealing is observed. As the (**c**) power factor is enhanced, (**d**) thermal conductivity is also reduced, giving *HQ* has an improved figure of merit, (**e**) zT, which decreases with low temperature annealing, as shown in samples *HQA8* and *HQA32*. Dashed lines are guides to the eye.

The room temperature physical properties for each sample are presented in Figure 7. The high temperature quenched sample, *HQ*, displays an enhanced Seebeck coefficient (a) and power factor (c), as well as a decrease in thermal conductivity (d). This leads to an overall increase in the thermoelectric figure of merit (e), zT. The enhancement of these properties decreases with annealing, or decreasing Ni interstitial abundance, as indicated by the trend line.

Ni-interstitials act as electron donors, decreasing the electrical resistivity of samples after the high temperature quench (Figure 7b). The increase in carrier concentration may be closer to optimal for these compounds, leading to a positive effect on the Seebeck coefficient [34]. The Seebeck coefficient increases in magnitude after the high temperature quench and is maximized where the amount of Ni-interstitials is maximized (Figure 7a). Calculations indicate that Ni-interstitials or antisite defects where Ni sits on the interstitial site cause “in-gap states”, leading to an experimental band gap of 0.12 eV, which is much smaller than the calculated band gap of 0.45 eV [27,35,42,43]. The Ni interstitials modify the density of states at the Fermi level, improving the Seebeck coefficient [32,44,45,46]. This interpretation follows the results presented earlier in this contribution, as they describe an increase in Ni interstitials for the quenched sample and a decrease upon subsequent annealing. The magnitude of the Seebeck coefficient decreases as Ni interstitials are annealed out. The phase separation present in the annealed samples could have a carrier filtering effect, selectively scattering low energy carriers due to the interfacial potential between the full- and half-Heusler phases [45].

The electrical properties of these samples are summarized by the thermoelectric power factor in Figure 7c. Due to its enhanced Seebeck coefficient, the high temperature quenched sample (*HQ*) has the largest power factor. While subsequent annealing decreases the power factor back towards that of the homogenized sample, the decrease in electrical resistivity due to these heat treatments leads to an overall enhancement of the power factor compared to the homogenized sample.

Nickel interstitials also have a strong role in determining thermal conductivity, shown in Figure 7d. As Ni-interstitials are introduced, they act as point defect phonon scattering centers, decreasing the lattice contribution to thermal conductivity [3]. The introduction of Ni interstitials due to the high temperature heat treatment and quench reduced the thermal conductivity by almost a factor of two, from 3.86 W m${}^{-1}$ K${}^{-1}$ to 2.00 W m${}^{-1}$ K${}^{-1}$. While these measurements were conducted at 300 K, the contribution from point defects should increase with temperature due to the shortening mean free path of phonons. Low temperature annealing decreases the abundance of Ni-interstitials, and the thermal conductivity increases. However, the microstructure, which consisted of micron-scale precipitates in the homogenized sample, becomes nanostructured due to phase separation after the high temperature quenching and low temperature annealing. This may lead to a permanent decrease in the overall thermal conductivity, as compared to the homogenized sample. There is ample evidence in the literature for increases in zT due to nano- and micro-scale phase separation in half-Heuslers [11,22].

The zT of these materials has been calculated, as shown in Figure 7e. Due to the impact of Ni-interstitials on both the Seebeck coefficient and the thermal conductivity, the quenched sample (*HQ*) has the greatest room temperature zT = 0.26, over five-times greater than zT of the homogenized sample (zT = 0.05). Further annealing of these samples decreases the quantity of Ni-interstitials, but leads to nanoscale Heusler precipitates within the half-Heusler matrix, giving an increase of over two-fold in the room temperature zT after 32 hours of low temperature annealing compared to homogenized TiNi${}_{1.1}$Sn.

## 4. Conclusions

In this contribution, samples of TiNi${}_{1.1}$Sn were heat treated to determine the effect of Ni interstitials on the physical properties. All samples were first homogenized in the biphasic regime, phase separating the full- and half-Heusler. Next, samples were quenched from a high temperature in the solid solution regime between TiNiSn and TiNi${}_{2}$Sn, and the final samples were annealed at low temperature for different lengths of time. The effect of processing on the prevalence of Ni interstitials was determined using synchrotron XRD and EDS, showing that when quenched from the solid solution regime, excess Ni was present in the half-Heusler TiNiSn tetrahedral vacant sites. These observations were related to thermoelectric physical properties, showing that the Ni-interstitials enhance the Seebeck coefficient, while simultaneously reducing thermal conductivity, resulting in an improved room temperature zT by at least a factor of five. Subsequent annealing reduces the Ni-interstitials and reforms TiNi${}_{2}$Sn at the nanoscale, which displays enhanced thermoelectric properties compared to the homogenized sample due to nanostructuring. While Ni interstitials in TiNi${}_{1.1}$Sn would be annealed out over time at the operating temperature of this thermoelectric, this study showed that the disorder from interstitial point defects has a positive effect on the thermal conductivity and Seebeck coefficient and results in significantly higher figures of merit. In addition, the heat treatment process to form a solid solution and re-precipitate out the Heusler phase provides a route to nanostructuring of the bulk half-Heusler with excess Ni and increasing the figure of merit with respect to homogenized TiNi${}_{1.1}$Sn.

## Figures and Tables

**Table 1:** Heat treatments for each sample are given. Values for goodness of fit ($R_{wp}$), mole percent of the full-Heusler phase and the weighted average lattice parameter of the half-Heusler phases (hH $a_{av}$) with Ni interstitials are determined from the Rietveld refinement of synchrotron XRD data.

Sample	Treatment	$R_{\mathrm{wp}}$ (%)	fHmol%	hH $a_{\mathrm{av}}$ (Å)
*H*	1173 K 144 h	9.75	12.7	5.918(2)
HQ	1173 K 144 h	12.0	8.6	5.969(1)
	1423 K 6 h + *Q*			
HQA8	1173 K 144 h	13.1	7.8	5.948(3)
	1423 K 6 h + *Q*			
	623 K 8 h			
HQA32	1173 K 144 h	15.4	8.8	5.947(2)
	1423 K 6 h + *Q*			
	623 K 32 h			
